# Glutamine Randomized Studies in Early Life: The Unsolved Riddle of Experimental and Clinical Studies

**DOI:** 10.1155/2012/749189

**Published:** 2012-09-18

**Authors:** Efrossini Briassouli, George Briassoulis

**Affiliations:** ^1^First Department of Propaedeutic Medicine, National and Kapodistrian University of Athens, Athens, Greece; ^2^Pediatric Intensive Care Unit, Division of Mother and Child, School of Medicine, University of Crete, Heraklion, Greece

## Abstract

Glutamine may have benefits during immaturity or critical illness in early life but its effects on outcome end hardpoints are controversial. Our aim was to review randomized studies on glutamine supplementation in pups, infants, and children examining whether glutamine affects outcome. Experimental work has proposed various mechanisms of glutamine action but none of the randomized studies in early life showed any effect on mortality and only a few showed some effect on inflammatory response, organ function, and a trend for infection control. Although apparently safe in animal models (pups), premature infants, and critically ill children, glutamine supplementation does not reduce mortality or late onset sepsis, and its routine use cannot be recommended in these sensitive populations. Large prospectively stratified trials are needed to better define the crucial interrelations of “glutamine-heat shock proteins-stress response” in critical illness and to identify the specific subgroups of premature neonates and critically ill infants or children who may have a greater need for glutamine and who may eventually benefit from its supplementation. The methodological problems noted in the reviewed randomized experimental and clinical trials should be seriously considered in any future well-designed large blinded randomized controlled trial involving glutamine supplementation in critical illness.

## 1. Introduction

Amino acids have a crucial role in protein synthesis, trigger signaling cascades that regulate various aspects of fuel and energy metabolism, and serve as precursors for important substrates. Glutamine, the most abundant amino acid in the muscle and plasma of humans traditionally considered a nonessential amino acid, now appears to be a conditionally essential nutrient during stress, injury [1], or illness [2]. During the acute stress of critical illness, large amounts of glutamine are produced by glutamine synthetase from muscle tissue [3] in response to stress and the regulation of glutamine synthetase protein turnover in response to glutamine concentrations [4]. Despite this significant release of glutamine, plasma levels decrease significantly following major burns in adults and remain decreased for over 21 days [5]. This severe glutamine deficiency occurs rapidly in adults and is associated with increased critical illness morbidity and mortality [6]. Similarly, the sudden cessation of glutamine supply from the mother to premature infants, who are already stressed and undergoing rapid growth, may be detrimental [7]. Thus, whereas plasma glutamine increases during the first days of life in breastfed infants [8], glutamine and arginine deficiencies have been reported in neonates suffering from acute illness [9]. In contrast to the breast milk containing glutamine adequate to influence gastrointestinal development and modulate immune, metabolic, and inflammatory responses of the newborn [10], standard infant formulas are low, and parenteral amino acid formulas are free of glutamine [11]. 

One described hypothesis for the release of glutamine following stress is that it provides a vital fuel source for rapidly dividing cells such as those of the immune system [12] and gastrointestinal tract [13], reticulocytes [14], and fibroblasts [15]; it is a precursor for nucleic acid synthesis, hexosamines, and nucleotides [12]; the nitric oxide precursor arginine [16]; the major antioxidant-glutathione [17]; a key precursor for acid-base homeostasis in the kidney [18]. In addition to its role as a gluconeogenic substrate in the intestine, liver, and kidney [19, 20], glutamine is involved in nitrogen transport from muscle to gut, kidney, and liver [21] and the regulation of acid-base homeostasis by renal regulation of interorgan glutamine flow in metabolic acidosis [22]. 

Glutamine may have benefits during experimental neonatal endotoxemia and in premature infants of very low birth weight (VLBW), who are highly stressed and have low energy and protein reserves [23]. Undergoing rapid growth, preterm infants are most likely to be exposed to severe glutamine deficiency (conditionally essential) more than term infants [7]. Glutamine supplementation may also be beneficial for critical childhood conditions including cancer, severe burns/trauma, as well as gastrointestinal disease and malnutrition. Its effects on pediatric systemic inflammation or acute illness, however, are unknown since less data is available on the effects of supplemental glutamine in infants and critically ill children. The administration of glutamine nutrient-enriched diets did not change the mortality of the critically ill or surgical adult patients, but infection complications in the critically ill patients, particularly the surgical population, were reduced [24].There is little information on the role of pharmaconutrients in neonatal endotoxemia, or whether glutamine supplementation is beneficial in preterm babies or critically ill children. The purpose of this paper was (a) to discuss the recent research-based evidence of the glutamine role in sepsis, which best of all describes a critical illness situation, and (b) to present the heterogeneity and differences of methods and results of randomized experimental and clinical studies of dietary glutamine in early life: from VLBW and preterm infants to children and up to adults.

## 2. The Glutamine Role in Sepsis Immunopathology and Metabolism

Sepsis is the systemic inflammatory response associated with an infectious insult. It is the leading cause of death in critically ill patients, and the predominant cause of multiple organ dysfunctions that is known to develop in response to infection. Sepsis occurs in over 750,000 patients per year in the United States and exhibits a mortality rate of 28% to 48% depending on the age of the patient [25]. Nosocomial infection/sepsis occurs in more than 40% of children requiring long-term intensive care [26]. Potential antiendotoxin strategies may have the potential to reduce severity of illness and length of Pediatric Intensive Care Unit (PICU) stay in critically ill children [27]. In very preterm (<32 weeks of gestation) and/or very low birth weight (VLBW < 1500 g birth weight) infants, serious neonatal infections are among the main causes of poor developmental outcomes later in childhood. Implementation of an emergency department septic shock protocol and care guideline improved compliance in delivery of rapid, aggressive fluid resuscitation and early antibiotic and oxygen administration and was associated with decreased length of stay [28]. Despite significant advances in critical care, there is still no efficient causal therapy applicable to patients indicating the need to further elucidate the molecular pathways leading to the immunopathology of sepsis [29]. In addition, host genetic variability in the regulatory and coding regions of genes for components of the innate immune system may influence the susceptibility to and/or outcome from sepsis [30]. 

Specific nutrients known as pharmaconutrients have demonstrated an ability to modulate the immunologic and inflammatory responses in clinical and laboratory studies. Among these substrates, arginine, glutamine, n-3 fatty acids, and nucleotides are the most relevant and exhibit the greatest immune-modulatory action [31]. Although the mechanisms of how pharmaconutrients benefit critically ill patients have not been established, experimental work has shown that glutamine regulates the expression of many genes related to signal transduction, antioxidant capacity, immune and metabolic function [32], protein synthesis, and degradation and activates intracellular signalling pathways [33]. Thus, the effectiveness of glutamine in preventing liver damage in neonatal sepsis appears to be mediated via glutathione synthesis [34]. In addition, glutamine could improve insulin sensitivity and glucose disposal in patients suffering from critical illness, a condition frequently associated with insulin resistance and subsequent hyperglycemia [35]. By being involved in the biosynthesis of hexosamines, glutamine maintains gut wall integrity via surface mucin and glycoprotein-forming intracellular tight junctions and protects against bacterial translocation [36]. When hepatocytes from endotoxaemic rats were incubated with glutamine, there was a restoration of mitochondrial structure and metabolism. In vivo, intraperitoneal injection of glutamine into endotoxic suckling rats partially reversed hypometabolism, markedly reduced the incidence of hypothermia, and improved clinical status [37]. 

Activation of NF-*κ*B is dependent on the phosphorylation and degradation of IKB-*α*, an endogenous inhibitory molecule that binds to NF-*κ*B in the cytoplasm [38]. In an experimental study glutamine suppressed NF-*κ*B transcriptional activation and translocation to the nucleus and significantly inhibited IKB-*α* phosphorylation and degradation in lung cytosolic tissue [39]. Similarly, glutamine administration attenuated the peak in IL-18 release at 12–24 h and maintained IL-18 levels at a consistently low level throughout the initial 24-h period after cecal ligation and puncture (CLP) in rats [39]. Thus, glutamine's effect on IL-18 expression may be an additional mechanism by which glutamine improves survival and decreases end-organ dysfunction following polymicrobial sepsis.

## 3. The Secrets of Molecular Chaperones 

The heat shock response is a highly conserved cellular mechanism that protects against injury and environmental stresses. Intracellular heat shock proteins (hsps) function as molecular chaperones governing protein assembly, folding, or transport and as anti-apoptotic regulators of cell signalling pathways leading to cell death [40]. In addition, hsp peptides promote the production of anti-inflammatory cytokines, indicating immune-regulatory potential of hsps [40]. Of particular importance is the expression of members of the 70-kd hsp70 family. Experimental results indicate that the expression of inducible hsp70 is vital to protect against the proinflammatory response and lung injury associated with sepsis. Induction of hsp60 has also been demonstrated in cerebral ischemia models, possibly reflecting mitochondrial stress and occurred early after the injury [41]. Sepsis, endotoxin tolerance, and heat shock all display downregulation of innate immunity, sharing a common immune suppressive effect, possibly through HS factor 1 (HSF-1)-mediated competitive inhibition of NF-*κ*B binding [42]. Heat shock protein 70 plasmid-transfected cells had increased hsp70 expression and demonstrated decreased nitric oxide (NO) release and inducible NO synthase messenger RNA expression in response to endotoxin compared with wild-type and empty plasmid-transfected cells [42]. Cell cycle components, regulatory proteins, and proteins in the mitogenic signal cascade may be protected by hsp70 during periods of stress [43]. 

Experimental evidence suggests that hsp70 expression is required for glutamine's protection against tissue injury and for attenuation of NF-*κ*B activation and proinflammatory cytokine release [44]. In the human gut, enteral glutamine may attenuate ubiquitin-dependent proteolysis as demonstrated by decreased ubiquitin RNA [45], and in lung and muscle, it can regulate glutamine synthetase protein degradation by facilitating its degradation by the 26S proteosome [46]. It was shown that absence of hsp70 alone can significantly increase ARDS, activation of NF-*κ*B, and inflammatory cytokine response whereas the specific absence of hsp70.1/3 gene expression can lead to increased mortality after septic insult [47]. The survival-promoting effects of hsp70 could also be attributed in part to the suppression of apoptosis, since reduced hsp expression in glutamine-deprived cells together with their impaired antioxidant capacity may make them more susceptible to apoptosis [48].

## 4. Glutamine Is a Prochaperone

A significant body of preexisting literature has hypothesized a relationship between hsp70 expression and glutamine's protection in both in vitro and in vivo settings [39–44]. Glutamine has been shown to induce heat shock protein expression and to attenuate lipopolysaccharide (LPS)-mediated cardiovascular dysfunction. The molecular mechanism of glutamine-induced hsp70 expression appears to be mediated via enhancement of O-linked *β*-N-acetylglucosamine (O-GlcNAc) modification and subsequently to increase levels of endonuclear HSF-1 expression and HSF-1 transcription activity [49]. The molecular mechanism of glutamine-mediated hsp70 expression appears to be dependent on O-GlcNAc pathway activation and subsequent O-glycosylation and phosphorylation of key transcription factors required for hsp70 induction [50]. It has been demonstrated that a single dose of intravenous glutamine enhances phosphorylation of nuclear HSF-1, which is a vital step in its transcriptional activation [51], causing a rapid and significant increase in hsp25 and hsp 72 expression in multiple organs of the unstressed Sprague-Dawley rat [52]. 

Pioneer studies showed that glutamine supplementation could attenuate lethal heat and oxidant injury and increase hsp72 expression in intestinal epithelial cells (IEC-6 cells) [53–55]. The effect of glutamine in delaying spontaneous apoptosis in neutrophils and protecting activated T cells may be mediated by upregulating glutathione [56] and Bcl-2 expression and inhibiting Fas [57]. Glutamine effectively improved vascular reactivity by inducing the expression of hsp70, reducing inflammatory cytokine release and peroxide biosynthesis in LPS shock rats [58]. In a recent study, septic mice with glutamine administration showed less severe damage to the kidneys and exhibited decreased high mobility group box protein-1 (HMGB-1), toll-like receptor-4, receptor of advanced glycation end-products (RAGE), and reduced nitrotyrosine levels in kidney tissues [59]. In glutamine-treated rats, lung hsp70 and HSF-1-p expressions were enhanced, lung HMGB-1 expression and NF-*κ*B DNA-binding activity were suppressed, ARDS was attenuated and survival improved [60]. Similarly, by inducing hsp70 in an experimental model, glutamine was also shown to attenuate LPS-induced cardiomyocyte damage [49]. 

Marked attenuation of tissue metabolic dysfunction was observed after glutamine administration as measured by lung tissue adenosine 5′-triphosphate/adenosine 5′-diphosphate ratio and the oxidized form of nicotinamide adenine dinucleotide [61]. Furthermore, the ATPase cycle of the chaperone hsp70 is regulated by co-chaperones. Hsp40 related proteins stimulate ATP hydrolysis by hsp70, whereas hsp25, which is known to be a vital protective protein via interaction with the cytoskeleton, may play an important role in glutamine's cellular protection [40]. It was shown that glutamine could protect intestinal epithelial cells in a dose-dependent fashion against heat stress and oxidant injury [53], decrease lung injury [61], and enhance hsp expression after endotoxin shock thus, improving survival [52]. It has also been shown that hsp70 levels increase in the myocardium of rats in experimental diabetes mellitus as a protective mechanism and may be further increased with parenteral administration of glutamine [62]. A randomized trial in adult patients with full-thickness burns showed for the first time that orally administered glutamine can enhance tissue hsp70 expression [63] and improve survival following lethal hyperthermia injury [64]. It was hypothesized that glutamine may act as a HSF-1 activator and increase the entire family of hsps after stress or injury since in HSF-1 knockout mouse fibroblasts, glutamine's ability to generate an hsp response is lost and the protection conferred by glutamine is also completely abrogated [51]. 

## 5. Ornithine: A Glutamine Alternative 

Glutamine is a substrate for polyamine synthesis and stimulates the activity of ornithine decarboxylase (ODC), a key enzyme for polyamine synthesis, in intestinal epithelial cells. In a recent experimental work, polyamines (putrescine, spermidine, or spermine) and their precursor ornithine mediated the induction of hsp expression in IEC-18 rat intestinal epithelial cells [65]. As previously observed, glutamine was required for heat stress-induction of hsp70 and hsp25, although it had little effect under basal conditions. Under conditions of glutamine depletion, supplementation of ornithine, or polyamines restored the heat-induced expression of hsp70 and hsp25. In the same study, when ODC was inhibited by *α*-difluoromethylornithine (DFMO), an irreversible ODC inhibitor, the heat stress-induction of hsp70 and hsp25 was significantly decreased even in the presence of glutamine [65]. Ornithine, polyamines, and DFMO did not modify the nuclear localization of HSF-1. However, DFMO dramatically reduced glutamine-dependent HSF-1 binding to an oligonucleotide with heat shock elements (HSE) which was increased by glutamine. In addition, exogenous polyamines recovered the DNA binding activity. These results indicated that polyamines play a critical role in the glutamine-dependent induction of the intestinal epithelial heat shock response through facilitation of HSF-1 binding to HSE [65].

## 6. Factors Influencing Glutamine Protective Role in Sepsis

Recent work demonstrated that febrile-range temperatures achieved during sepsis and noninfectious SIRS correlated with detectable changes in stress gene expression in vivo (whole blood messenger RNA), thereby suggesting that fever can activate hsp70 gene expression and modify innate immune responses [66]. In addition, analysis of septic patients according to survival outcome indicated that hsp70 serum levels were modulated according to the patient oxidant status [67]. Unexpectedly, hsp70 was shown to be a key determinant of mortality in aged, but not young hosts in sepsis. It might be concluded therefore, that hsp70 may play a protective role in an age-dependent response to sepsis by preventing excessive gut apoptosis and both pulmonary and systemic inflammation [68]. 

Critically ill patients display variable physiologic responses when stressed; gene association studies have recently been employed to explain this variability. Genetic variants of hsp70 have also been associated with the development of septic shock in patients [69, 70]. The specific absence of hsp70.1/3 gene expression can lead to increased mortality after septic insult [47]. 

Drug interactions were also shown to either suppress hsp protective effects exacerbating therefore drug-induced side effects or to induce hsp beneficial effects by suppressing drug-induced exacerbations. Thus, it was recently shown that bleomycin-induced pulmonary fibrosis is mediated by suppression of pulmonary expression of hsp70 whereas an inducer of hsp70 expression, such as geranylgeranylacetone, may be therapeutically beneficial for the treatment of gefitinib-induced pulmonary fibrosis [95]. 

## 7. Randomized Adult Studies

Despite the enthusiastic experimental results, suggesting a beneficial effect for the immune-enhancing glutamine, systematic reviews and meta-analyses of randomized studies failed to show any definite benefit of pharmacononutrition in the critically ill adult [96]. Most of the randomized studies have been largely performed in surgical patients [97], and only a few in trauma patients receiving glutamine-supplemented enteral nutrition [98] or septic patients on enteral immunonutrition [99].

In a multicenter, prospective, double-blind, randomized trial total parenteral nutrition (PN) supplemented with alanine-glutamine in intensive care unit patients was associated with a reduced rate of infectious complications and better glycemic control [100]. In another recent multicenter randomized, double blinded, factorial, controlled trial, 502 intensive care patients requiring PN were supported with parenteral glutamine (20.2 g/day) or selenium (500 *μ*g/day) or both, for up to seven days [101]. The primary (intention to treat) analysis showed no effect on new infections or on mortality when PN was supplemented with glutamine or selenium. Also, length of stay, days of antibiotic use, and modified SOFA score were not significantly affected by selenium or glutamine supplementation.

## 8. Randomized Experimental and Clinical Studies of Glutamine Supplementation in Early Life

The search methods for identification of studies consisted of searches of PubMed database using the search terms: “glutamine” and “critical illness,” or “sepsis,” or “endotoxemia.” The search output was limited with the search filter for ages: pups, infants, and children. References in selected studies were examined also. The title and abstract of all studies identified by the above search strategy were screened and the full text for all potentially relevant studies published in English was obtained. The full text of any potentially relevant studies was assessed by the two authors (EB, GB). The same authors extracted data from the published studies. Study populations, main methodological data and results on outcome are presented in Tables 1, 2, and 3.

### 8.1. Glutamine Supplementation in Animal Pups

Using light and transmission electron microscopy in artificially reared rat pups the greatest blunting of villus height in the ileum and the lowest number of villi per unit length of bowel were in the animals that were treated with inhibition of glutamine and not provided with dietary glutamine [75]. Transmission electron microscopy demonstrated breakdown of the epithelial junctions in the glutamine-deprived and glutamine synthetase-inhibited intestines. Glutamine-deprived animals also displayed sloughing of microvilli, decreased actin cores, and degeneration of the terminal web [75]. In an experimental controlled study, glutamine and leucine both caused nutrient-induced thermogenesis in control animals and restored oxygen consumption (VO_2_, mL/kg/h) of endotoxic suckling rats [71]. Glutamine additionally increased rectal temperature, reduced incidence of hypothermia, and improved clinical signs. Undernourished pups/dam supplemented with glutamine with or without zinc showed increased CA1 layer volume and hippocampal gamma-aminobutyric acid and synaptophysin levels as compared with the other groups, consistent with the trend toward increased number of neurons and protection against malnutrition-induced brain developmental impairments [72]. Also, glutamine supplementation during brain development facilitated cortical spreading depression propagation, as judged by the higher cortical spreading depression velocities, recorded on 2 cortical parietal points of the right hemisphere, an effect not abolished by malnutrition [73]. Importantly, when injected intraperitoneally in eleven-day rat pups, glutamine partially prevented the sepsis-induced fall in plasma glutamine levels and reduced the concentration of both proinflammatory and anti-inflammatory cytokines [74]. 

### 8.2. Parenteral Glutamine in Extremely Low Birth Weight (ELBW) and Preterm Infants

Glutamine appears to be safe for use in premature infants and seems to be conditionally essential in premature infants with extremely low birth weights [82]. In ELBW infants, parenteral glutamine supplementation could increase plasma glutamine concentrations without apparent biochemical risk [83]. When supplemented PN for more than 2 weeks, glutamine shortened days on PN and length of stay in hospital, and decreased hospital acquired infection episodes in premature infants [81]. Although parenteral glutamine failed to enhance rates of protein synthesis, it was assumed that it may have an acute protein-sparing effect, as it suppressed leucine oxidation and protein breakdown, in parenterally fed very low birth weight infants [80]. Thus, parenteral glutamine supplementation was shown to be associated with lower whole-body protein breakdown and protein accretion in selected populations of LBW infants [77] and to improve hepatic tolerance in VLBW infants, suggesting a hepato-protective effect [84]. In another randomized study, although parenteral glutamine appeared to be well tolerated and safe in the ill preterm neonate, able to reduce the time to achieving enteral nutrition, it did not reduce the episodes of culture-positive sepsis or age at discharge [78].

In a large multicenter, randomized, double-masked, clinical trial in ELBW infants, the safety and efficacy of early PN supplemented with glutamine in decreasing the risk of death or late-onset sepsis were assessed; infants 401 to 1000 g were randomized within 72 hours of birth to receive either TrophAmine (control) or an isonitrogenous study amino acid solution with 20% glutamine whenever they received PN up to 120 days of age, death, or discharge from the hospital [76]. Of the 721 infants assigned to glutamine supplementation, 370 (51%) died or developed late-onset sepsis, as compared with 343 of the 712 control infants (48%). Also glutamine had no effect on tolerance of enteral feeds, necrotizing enterocolitis, or growth. No significant adverse events were observed with glutamine supplementation. Accordingly, although no harm was demonstrated, parenteral glutamine supplementation as studied in this large study could not be recommended in ELBW infants [76]. In a double-blind, randomized trial, short-term glutamine-supplementation (0.4 g/kg/day) of PN did not show any benefit on intestinal permeability in newborns and infants after major digestive-tract surgery [79]. Similarly, in a recent randomized clinical trial of glutamine-supplemented versus standard PN, glutamine supplementation did not reduce the incidence of sepsis in surgical infants with gastrointestinal disease [85]. Thus, using the Cochrane Central Register of Controlled Trials (The Cochrane Library, 2011, Issue 4), MEDLINE, EMBASE and CINAHL (to November 2011), conference proceedings and previous reviews, it has been recently concluded that the available trial data do not provide evidence that glutamine supplementation confers important benefits for preterm infants [102]. 

### 8.3. Enteral Glutamine in ELBW and Preterm Infants

Double-blind randomized trials in VLBW infants did not show that enteral glutamine supplementation decreases morbidity or mortality [87, 103]. Blinded, randomized studies provided evidence for a blunting of the inflammatory process [86] and lower sepsis rates in VLBW infants receiving enteral glutamine supplementation [88]. In contrast to reports showing better tolerance to enteral feedings in glutamine-enriched enteral nutrition in VLBW infants [87], other studies did not find any improvement of the median days to reach full enteral feeds [86, 88]. In addition, despite a decreased infectious morbidity, glutamine supplementation in VLBW infants was not associated with alterations in the prevalence of *bifidobacteria*, *lactobacilli*, *E. coli* [89] and did not enhance the postnatal decrease in intestinal permeability in this population [90]. Importantly, examining the effect of enteral glutamine on whole-body kinetics of glutamine in growing preterm infants, enterally administered glutamine was shown to be entirely metabolized in the gut and to have no detectable effect on whole-body protein and nitrogen kinetics [104]. Studied by dual tracer cross-over techniques, dietary glutamine was shown to be used to a great extent by the splanchnic tissues in preterm infants, its carbon skeleton having an important role as fuel source [105]. Similarly no differences between groups for plasma concentrations of glutamine, glutamate, other amino acids, glucose, or ammonia were shown during the enteral supplementation of glutamine [106, 107].

Followup of all surviving VLBW infants having received enteral preterm formula or breast milk supplemented with glutamine showed a lower risk of atopic dermatitis but no differences in incidence of bronchial hyperactivity, infections of upper respiratory, lower respiratory, urinary, or gastrointestinal tracts [108, 109], of intestinal microbiota, neurodevelopmental impairment or cerebral palsy [110]. No difference was also detected among randomized groups in intestinal (faecal) microbiota at age 1 year as analyzed by fluorescent in situ hybridization [111] or in TH1 and TH2 cytokine profiles either during the days of supplementation [91] or at 1 year of age following in vitro whole blood stimulation [112]. Importantly, glutamine-enriched enteral nutrition in VLBW infants had neither beneficial nor detrimental effects on long-term cognitive, motor, and behavioral outcomes of very preterm and/or VLBW children at school age, although visuomotor abilities were poorer in children that received glutamine [113].

### 8.4. Effect of Glutamine Supplementation on Critically Ill Children

In a multicenter randomized, double-blinded, comparative effectiveness trial, zinc, selenium, glutamine, and intravenous metoclopramide conferred no advantage in the immune-competent population of children requiring long-term intensive care compared with whey protein supplementation [26]. Further evaluation of these constituents' supplementation was thought to be warranted only in the immune-compromised long-term pediatric intensive care unit patient. 

In a blinded, prospective, randomized, controlled clinical trial in critically ill children given an immune-enhancing formula supplemented with glutamine, a favorable effect on nutritional indices and antioxidant catalysts was reported, but none on outcome hard endpoints [92]. Also, although it posed a higher metabolic burden to the patient, it showed a trend to improve colonization and infection rates; diarrhea in the immunonutrition and gastric distention in the control group were the most frequently recorded complications [92]. In another single-center, randomized, blinded controlled trial in children with septic shock a significant decrease in IL-6 levels was recorded after 5 days of early enteral feeding using the same immune-enhancing formula [93]. The variation in cytokines was independently correlated only to PRISM, but mortality and other pediatric intensive care unit outcome hard endpoints did not differ between the two groups. Similarly, in another randomized, blinded, controlled study in children with severe head injury, using masked isocaloric formulae, immunonutrition improved nitrogen balance and decreased interleukin-8 and gastric colonization but was not associated with additional clinical advantage over the one demonstrated by conventional early enteral nutrition [94]. In two randomized studies in trauma patients (children and adults) no significant differences were also recorded in mortality, LOS, lung infection or immunologic or biochemical parameters between the glutamine supplemented groups (enteral or parenteral) and controls [114, 115].

## 9. Concluding Remarks

Given the encouraging experimental results, the absence of glutamine-related adverse effects, and the immune cells need for glutamine to grow and multiply, it has long been hypothesized that high risk patients could benefit from the use of this highly promising pharmaconutrient [12]; furthermore, combining glutamine with antioxidants and other selective pharmaconutrients, might exert an extra-synergic immune-enhancing effect. Thus, parenteral glutamine supplementation for 7 days increased total plasma glutathione levels in adult trauma patients receiving standard enteral nutrition [116]. In another study, however, glutamine administration by enteral or parenteral routes did not appear to affect antioxidant capacity or oxidative stress markers compared to unsupplemented adult ICU patients [117]. 

Studies evaluating the effect of specific pharmaconutrients in premature infants and critically ill children are scarce and are insufficient to allow recommendations to be made. Recent evidence from studies in various pediatric diseases and in premature newborns remains inconsistent, partly because of the different effects of enteral and parenteral glutamine supplementation, inefficient treatment doses [118], co-administered multi-immune-enhancing constituents or more or less amino-acids, heterogeneity, timing, dosing regimens, and other [119]. In our meta-analysis, none of the randomized studies in early life showed any effect in mortality, one in the length of stay, and only a few showed some effect on inflammatory response, organ function and a trend for infection control. Thus, although apparently safe in ELBW premature infants and in critically ill children, blinded controlled randomized studies in these populations concluded that glutamine supplementation does not reduce mortality or late onset sepsis, and its routine use cannot be recommended for the immune-competent patients.

Once again, laboratory research preliminary beneficial effects failed to be reproduced in clinical randomized (and/or blinded and masked) studies. The lack of beneficial effect of glutamine supplementation in sepsis might be explained by any of the factors influencing glutamine protective role as described above. Various factors during the clinical course might have also influenced results of those studies. In addition, by using isonitrogenous controls (to ensure the specific effect of glutamine), the overall amino acid intake may have been inadequate in the glutamine group, as a consequence of the substitution of 20% of the standard amino acids with glutamine. Although adding more amino acids to the control groups might have prevented the removal of amino acids from the glutamine supplement, the isonitrogenous control groups would have then received more amino acid/nitrogen than the recommended allowances. On the other hand, enteral supplementation of glutamine seems to be locally used by the intestine but may not help entering the systemic circulation to enhance the immune response. All these different research methods along with the use of different feeding guidelines for the introduction or withholding parenteral and enteral feeds among institutions make comparisons between studies difficult [120]. Further molecular and biochemical data is needed along with large randomized controlled trials in select populations of sick children, such as immune-compromised, who may eventually benefit from supplemental glutamine.

## Figures and Tables

**Table 1:** Methods and results of randomized, controlled studies investigating potential beneficial effects of glutamine supplementation in mortality, morbidity, hospital acquired infections, length of stay, or inflammation in endotoxic neonatal animals.

Animal models (pups)	*n*	Combined with other immunonutrients or inducers	Dose	Route	Duration	Mortality	Hospital- acquired infections	Length of stay	Organ function/Morbidity	Inflammation
Endotoxic 11–13-day-old Wistar rat pups [71]	5	Saline plus LPS plus glutamine	2 mol/kg	Single intraperitoneal injection	90–210 min	—	Improved clinical signs of endotoxic rats	—	—	Restored VO_2_ of endotoxic animals

Undernourished swiss mice pups/dam [72]	12	Zinc acetate was added in the drinking water (500 mg/L) to the lactating dams	100 mM, 40–80 microL	Daily supplementation with subcutaneous injections	2–14 days	—	—	—	Protects against malnutrition-induced brain developmental impairments	—

Male Wistar suckling rat pups, well-nourished and malnourished during lactation [73]	6–12	No	500 mg/kg/day	By gavage during postnatal days 7 to 27	7 to 27 days	—	—	—	In both nutritional condition, Glutamine rats presented higher cortical spreading depression propagation as compared to water-treated controls	—

Eleven-day rat pups [74]	7–10	Saline plus 300 microg/g *Escherichia coli* lipopolysaccharide	2 mmol/g	Intraperitoneal injections glutamine	2 or 6 hours	—	—	—	—	TNF*α*, IL-10 increased by endotoxemia were partly prevented by glutamine

Artificially reared 11 to 13-day-old Wistar rat pups [75]	30	Groups with inhibition of glutamine synthetase by methionine sulfoximine	40 g/kg per day total protein, 10 to 15% of which is glutamine + glutamate, added to a mixture containing carbohydrates, lipids, and vitamins	Artificial feeding using the rat infant “pup in the cup” model through gastrostomy	7–11 days	—	—	—	Glutamine-deprived animals demonstrated breakdown of the epithelial junctions, sloughing of microvilli, decreased actin cores, and degeneration of the terminal	—

LPS: lipopolysaccharide; IL: interleukin; TNF*α*: tumor necrosis factor alpha; NS: nonstatistical difference.

**Table:** (a)

Premature or ELBW infants on parenteral nutrition	*n*	Combined with other immunonutrients or inducers	Dose	Route	Duration	Mortality	Hospital- acquired infections	Length of stay	Organ function/Morbidity	Inflammation
Extremely low birth weight infants [76]	1433	No	Isonitrogenous study amino acid solution with 20% glutamine	Early parenteral nutrition	120 days	NS	NS	NS	Increased plasma Glutamine concentrations but also more days of PN support. No differences of late onset sepsis, NEC, day to first and full enteral feeds, feeding intolerance, or growth	—

Premature infants ≤32 weeks gestation with a birth weight from 694 to 1590 g [77]	20	No	0.6 g/kg/day	Early parenteral nutrition with amino acid intake 3.0 g/kg/day for at least 3 days	Tracer isotope studies at 6 to 7 days old	—	—	—	Supplemental glutamine was associated with a lower rate of appearance of glutamine, phenylalanine, and leucine C. No difference in leucine N and urea turnover	No significant difference in plasma cortisol and C-reactive protein levels

Ill preterm neonates of <1000 g birth-weight [78]	35	No	16% of the total amino acids (amino acids 1–3.0 g/kg/day)	Early parenteral nutrition	For 7 days or more	—	NS	NS	No significant differences between the groups in blood urea nitrogen, plasma ammonia, plasma glutamine, or glutamate	No significant differences in white cell count, differential white cell count, lactate, pyruvate

Infants after major digestive-tract surgery [79]	41	No	0.4 g/kg/day	Early parenteral nutrition	1–4 weeks	NS	NS	NS	—	—

VLBW age < 3 d, birth wt: 820–1650 g; GA: 28–30 wk [80]	13	No	0.5 g/kg/day	Exclusive parenteral nutrition	Day 4 of life for 24 hours	—	—	—	Decreased rates of Leu release from protein breakdown and Leu oxidation, decreased rates of nonoxidative Leu disposal (an index of whole-body protein synthesis), safe	—

Premature infants [81]	53	No	Isonitrogenous study amino acid solution with 20% glutamine	Early parenteral nutrition	14 days	NS	Lower	Shorter	Fewer days on PN, regained birth weight sooner	—

VLBW premature neonates age < 4 d receiving PN for <3 d; birth wt: 530–1250 g; GA < 32 wk [82]	44	No	15–25% of amino acid mix	Early parenteral nutrition	14 ± 6 days	—	—	NS	Birth wt < 800 g subgroup fewer d on PN, fewer d to full feeds, fewer d on ventilator, safe	Higher lymphocyte count

Infants with birth weights of 401–1000 g [83]	141	No	Isonitrogenous amino acid solution with 20% of the total amino acids as glutamine	Parenteral glutamine supplementation on plasma amino acid concentrations	10 days	—	—	—	No significant difference between the 2 groups in the relative change in plasma glutamate concentration but significant decreases in plasma phenylalanine and tyrosine between the baseline and PN samples	

VLBW Infants [84]	30	No	0.3 g/kg/day	Early parenteral nutrition	For ≥7 days	NS	NS	NS	No differences in time to full EN, episodes of gastric residual, total duration of PN, weight gain; hepatic function improved	—

Surgical infants less than 3 months old who required parenteral nutrition [85]	174	No	0.6 g/kg/day or isonitrogenous isocaloric parenteral nutrition (control group)	Early parenteral nutrition	Until full enteral feeding	NS	NS	—	No difference in time to full enteral feeding or time to first enteral feeding	—

**Table:** (b)

Premature or ELBW infants on enteral nutrition	*n*	Combined with other immunonutrients or inducers	Dose	Route	Duration	Mortality	Hospital- acquired infections	Length of stay	Organ function/Morbidity	Inflammation
VLBW age < 3 d receiving PN; birth wt: 500–1250 g; GA: 24–32 wk [86]	68	No	0.08 g/kg/d on d3 and reached 0.31 g/kg/d by d13	Glutamine-enriched enteral nutrition (PN *n* = 35)	Day 3–30 of life	NS	Reduced hospital-acquired sepsis (positive blood culture)	NS	No differences in NEC, wt, length, head circumference, mechanical ventilation, safe	Blunted the rise in HLA-DR+ and CD16/CD56 subsets

Critically ill infants 1–24 mo tolerating EN [87]	9	No	0.3 g/kg/day	Glutamine-enriched enteral nutrition	5 days	NS	NS	NS	Well tolerated and safe	—

VLBW age < 7 d receiving PN; birth wt: 500–1250 g [87]	649	No	0.3 g/kg/day	Within the first 7 d of age, randomly assigned to enteral glutamine supplement (3% glutamine in sterile water) or placebo (sterile water) given at the same time but separate from feedings	7 days–36 weeks post menstrual age	NS	NS	NS	Less gastrointestinal dysfunction, severe neurological sequelae among survivors (grades 3 and 4 intraventricular hemorrhage and paraventricular leukomalacia) in glutamine group. No difference in NEC, retinopathy of prematurity, oxygen use at 36 weeks, or growth,	—

VLBW infants < 48 h after birth receiving PN; birth wt: <1500 g; GA < 32 wk [88]	102	No	Increasing doses from day 3–30 of life to a maximum dose of 0.3 g/kg/day	Glutamine-enriched isonitrogenous enteral nutrition added to breast milk or preterm formula	Day 3–30 of life	NS	Lower incidence of ≥1 serious infections	NS	No difference in feeding tolerance, NEC, or growth, patent ductus arteriosus, mechanical ventilation, supplemental oxygen, retinopathy	—

VLBW < 48 h after birth receiving PN; birth wt: <1500 g; GA < 32 wk [89]	86	No	Increasing doses to ≤0.3 g/kg/day	Enteral preterm formula or breast milk supplemented with Glutamine or isonitrogenous Ala	Day 3–30 of life	NS	NS	—	No difference in prevalence of intestinal microflora (bifidobacteria, lactobacilli, *Escheria coli*, streptococci, clostridia) at <48 h–d30 of life, by fluorescent in situ hybridization	—

VLBW < 48 h after birth receiving PN; birth wt: <1500 g; GA < 32 wk [90]	90	No	Increasing doses to ≤0.3 g/kg/day	Enteral preterm formula or breast milk supplemented with glutamine or isonitrogenous Ala	Day 3–30 of life	NS	NS	—	No difference in decreased lactulose/mannitol ratio or urinary lactulose or increased urinary mannitol concentrations	—

VLBW infants <48 h after birth receiving PN; birth wt: < 1500 g; GA < 32 wk [91]	63	No	Increasing doses to ≤0.3 g/kg/day	Enteral preterm formula or breast milk supplemented with glutamine or isonitrogenous Ala	Day 3–30 of life	NS	NS	—	—	No differences in Th1 or Th2 cytokine responses at 48 h–d 14 of life following in vitro whole blood cell stimulation

ELBW: extremely low birth weight; VLBW: very low birth weight; Wt: weight; GA: gestational age; AA: amino acid; PN: parenteral nutrition; EN: enteral nutrition; LOS: length of stay; NEC: necrotizing enterocolitis; NB: nitrogen balance; IL: interleukin; NS: nonstatistical difference.

**Table 3:** Methods and results of randomized, controlled studies investigating potential beneficial effects of glutamine supplementation in mortality, morbidity, hospital acquired infections, length of stay, or inflammation in critically ill children.

Critically ill children	*n*	Combined with other immunonutrients or inducers	Dose	Route	Duration	Mortality	Hospital- acquired infections	Length of stay	Organ function/ Morbidity	Inflammation
Long-term intensive care patients (age 1–17 yrs) expected to require >72 hrs of invasive care [26]	293	Nutriceutical supplementation with zinc, selenium, glutamine, and metoclopramide (a prolactin secretalogue) compared to whey protein	0.3 g/kg/day	Glutamine by feeding tube each morning	Daily for up to 28 days	NS	Reduction in the immune-compromised group	NS	NS	—

Critically ill children [92]	50	L-arginine, docosahexaenoic eicosapentaenoic acid, selenium	1.04 g/100 kCal	Early enteral feeding	5 days	NS	Trend for less	NS	Higher osmolality, sodium, urea. Diarrhea and gastric distention the most frequently recorded complications	Increased NB

Children with septic shock [93]	40	L-arginine, docosahexaenoic eicosapentaenoic acid, selenium	1.04 g/100 kCal	Early enteral feeding	5 days	NS	NS	NS	NS	Decreased IL-6

Children with severe head injury [94]	38	L-arginine, docosahexaenoic eicosapentaenoic acid, selenium	1.04 g/100 kCal	Early enteral feeding	5 days	NS	NS	NS	NS	Decreased IL-8

NB: nitrogen balance; IL: interleukin; NS: nonstatistical difference.
